# Homœopathic Therapeutics of Diarrhœa, Etc.

**Published:** 1888-10

**Authors:** 


### BOOK NOTICES.

The Homœopathic Therapeutics of Diarrhœa, Dysentery, Cholera, Cholera Morbus, and Cholera Infantum, etc. By James B. Bell, M. D. Third edition. Philadelphia: F. E. Bœricke. The Hahnemann Publishing House. 1888.

The first edition of this valuable book was issued in 1869, and since that date the work has been a standard authority in all that pertains to the therapeutics of diarrhœa and dysentery. Having been so long before the medical public it needs no encomiums to enhance its value; the work is one which no homœopathic physician can afford to be without. It has been the means of saving very many lives. No greater commendation than this can be given any medical work.

An especially valuable feature of it is the clinical advice given after the therapeutics of each remedy. These notes are brief, clear-cut, and reliable. They have, doubtless, greatly aided many a physician in times of doubt and perplexity. This new edition contains the therapeutics of five new remedies. Four remedies, given in the previous edition, are omitted, viz., Cactus, Castoreum, Euphorbium, and Opuntia. Had running headings been used the work could have been consulted the easier. If an addition may be allowed, we would note here a symptom of Croton-tigl., which is peculiar to that remedy in diarrhœa. The characteristics of Croton are the yellow, watery stool, its sudden, quick discharge and prompt aggravation after taking food or drink; add to these the peculiar "sensation as of water swashing in the abdomen" whenever moving. Another comment: on page 175, we read: "Urination only possible with stool," which means only possible when straining, as at stool; this is the correct symptom. To pass urine the patient must strain as though he would have a stool. On page 125, we have another rendering of this symptom, and one we believe to be wrong. It is, "Expulsion (of stool) only possible when urinating."

In the preparation of this new edition Dr. Bell was assisted by Dr. S. A. Kimball, also of Boston.

